# Percutaneous Left Atrial Appendage Closure With a Novel LAA Occluder for Stroke Prevention in Atrial Fibrillation

**DOI:** 10.1016/j.jacasi.2022.04.009

**Published:** 2022-09-06

**Authors:** Xiaochun Zhang, Shiqiang Hou, Weijing Liu, Wei Chen, Fadong Chen, Wei Ma, Jian’an Wang, Youqi Fan, Yan Wang, Dong Chang, Hua Fu, Heng Cai, Yushun Zhang, Cody R. Hou, Yawei Xu, Daxin Zhou, Junbo Ge

**Affiliations:** a Department of Cardiology, Zhongshan Hospital, Fudan University, Shanghai Institute of Cardiovascular Disease, Shanghai, People’s Republic of China; b National Clinical Research Center for Interventional Medicine, Shanghai, People’s Republic of China; c Department of Cardiology, Shanghai Tenth People's Hospital, Tongji University School of Medicine, Shanghai, People’s Republic of China; d Department of Cardiology, Shanghai Tongji Hospital, Tongji University School of Medicine, Shanghai, People’s Republic of China; e Department of Cardiology, Peking University First Hospital, Beijing, People’s Republic of China; f Department of Cardiology, The Second Affiliated Hospital Zhejiang University School of Medicine, Zhejiang, People’s Republic of China; g Department of Cardiology, Xiamen Cardiovascular Hospital, Xiamen University, Fujian, People’s Republic of China; h Department of Cardiology, West China Hospital, Sichuan University, Sichuan, People’s Republic of China; i Department of Cardiology, Tianjin Medical University General Hospital, Tianjin, People’s Republic of China; j Department of Cardiology, First Affiliated Hospital of Xi 'an Jiaotong University, Shaanxi, People’s Republic of China; k College of Biological Sciences, University of Minnesota Twin Cities, Minneapolis, Minnesota, USA

**Keywords:** disc-like occluder, follow-up studies, stroke prevention, thrombosis, AF, atrial fibrillation, DRT, device-related thrombus, LAA, left atrial appendage, NVAF, nonvalvular atrial fibrillation, OAT, oral anticoagulation therapy, PDL, peridevice leak, SAE, serious adverse event, TEE, transesophageal echocardiography

## Abstract

**Background:**

More than 90% of thromboses originate from the left atrial appendage (LAA) in patients with nonvalvular atrial fibrillation (NVAF).

**Objectives:**

This study was designed to investigate the safety and efficacy of LAA closure with the Leftear device (Pulse Scientific) in NVAF patients.

**Methods:**

A prospective, multicenter, registry-based study was conducted in 200 NVAF patients with CHA_2_DS_2_-VASc (congestive heart failure, hypertension, age, diabetes, previous stroke/transient ischemic attack, vascular disease, female sex) scores ≥2. The primary safety endpoint was defined as any serious adverse events. Efficacy was assessed by a primary composite endpoint of hemorrhagic or ischemic stroke, systemic embolism, and cardiac or unexplained death at 1 year of follow-up.

**Results:**

The device was implanted in 196 patients, with 1-stop LAA closure combined with atrial fibrillation ablation implemented in 133 patients. The immediate success rate was 100%. There were serious adverse events in 9 patients (4.5%; 95% CI: 1.6%-7.4%), which mainly occurred in 1-stop LAA closure. All pericardial tamponades occurred in 6 patients with 1-stop LAA closure. No patient experienced a major bleeding event or acute device-related thrombus. During the 12-month follow-up period, the risk of the primary composite endpoint was 1.6% (95% CI: 0.3%-4.5%), and statistical noninferiority was achieved (the upper bound of 95% CI: 4.5% < the prespecified maximum annual incidence of 8.0%). Ischemic stroke occurred in 1 patient, 3 patients had incomplete LAA sealing, and no delayed device-related thrombus was found.

**Conclusions:**

LAA closure with the novel disc-like occluder shows high procedural success, satisfactory safety, and encouraging efficacy for stroke prevention in patients with NVAF. Compared with 1-stop LAA closure, single LAA closure may be more tolerable. (A multicenter, single-arm clinical trial to evaluate the efficacy and safety of left atrial appendage system for left atrial appendage occlusion in patients with non-valvular atrial fibrillation; ChiCTR1900023035)

Atrial fibrillation (AF) is a common arrhythmia, and its incidence increases with age.^1^ Thrombosis caused by AF is one of the main causes of stroke. Patients with nonvalvular atrial fibrillation (NVAF) have a 5-fold increased risk of stroke compared with patients with normal sinus rhythm caused by blood stasis caused by abnormal atrial beating. Oral anticoagulation therapy (OAT) is the current common and effective therapy used to prevent stroke associated with AF.^2^ Unfortunately, OAT is generally underused, and it is deemed not to be the ideal long-term treatment.

The left atrial appendage (LAA) is a common site of cardiac thrombosis. More than 90% of thromboses originate from the LAA in patients with NVAF.^3^^,^^4^ Since the 1930s, ligation of the LAA has gradually become a routine procedure in patients with heart disease and risk of left atrial thrombus.^3^, ^4^, ^5^ Surgical removal of the LAA in combination with ligation and endocardial suture significantly reduced the risk of ischemic stroke or systemic embolism.^6^ With the optimization of LAA closure device design and improvements in implantation technology, percutaneous LAA closure has been developed as an alternative strategy to OAT for stroke prevention in patients with NVAF.^2^^,^^7^ Several LAA closure devices were proven to have favorable safety and efficacy, including the WATCHMAN device (Boston Scientific) Amplatzer Cardiac Plug (St. Jude Medical), and LAmbre (Lifetech Scientific).^8^, ^9^, ^10^ Because of the large variability in the shape of the LAA, occlusion devices based on different principles are being continuously developed. The Leftear device (Pulse Scientific) is a new, fully recapturable and repositionable LAA occluder, specifically designed for LAA closure via the transseptal route into the LAA. At present, there are no data available on the clinical safety and efficacy of this device. This prospective, multicenter clinical study reported the initial experience and 1-year follow-up outcomes for implantation of the novel disc-like occluder in patients with NVAF in China.

## Methods

### Study population

This was a multicenter, nonrandomized, pilot trial designed to evaluate the safety, feasibility, and efficacy of using the novel disc-like occluder (A multicenter, single-arm clinical trial to evaluate the efficacy and safety of left atrial appendage system for left atrial appendage occlusion in patients with non-valvular atrial fibrillation; ChiCTR1900023035). The prospective study was conducted on 200 patients receiving percutaneous LAA closure in 9 hospitals in China from August 2018 to October 2019. All patients were diagnosed with NVAF, older than 18 years of age, scored at least 2 points according to the CHA_2_DS_2_-VASc (congestive heart failure, hypertension, age ≥75 years, diabetes, and previous stroke/transient ischemic attack, vascular diseases, female sex) score, and had at least 1 of the following: scored at least 3 points according to the HAS-BLED (hypertension, abnormal renal/liver function [1 point each], stroke, bleeding, labile international normalized ratios [INRs], elderly [age＞65 years], drugs and alcohol [1 point each]) score, or had poor effectiveness of OAT (stroke or thromboembolism on the basis of taking anticoagulant agents up to standard).^11^^,^^12^ All patients underwent transthoracic echocardiography and transesophageal echocardiography (TEE) before LAA closure. Patients with left atrial thrombosis, left ventricular ejection fraction <35%, or LAA orifice diameter <10.5 mm or >35 mm, or LAA depth <10 mm were excluded from the study. Other exclusion criteria included occasional AF, acute myocardial infarction within 3 months, prior stroke or transient ischemic attack within 30 days, hemorrhagic disease, presence of a prosthetic valve, or presence of an atrial septal repair or closure history.^8^ All subjects obtained informed consent and the study was approved by the institutional review committee of Zhongshan Hospital, Fudan University.

### Device implantation

**Figure 1:**
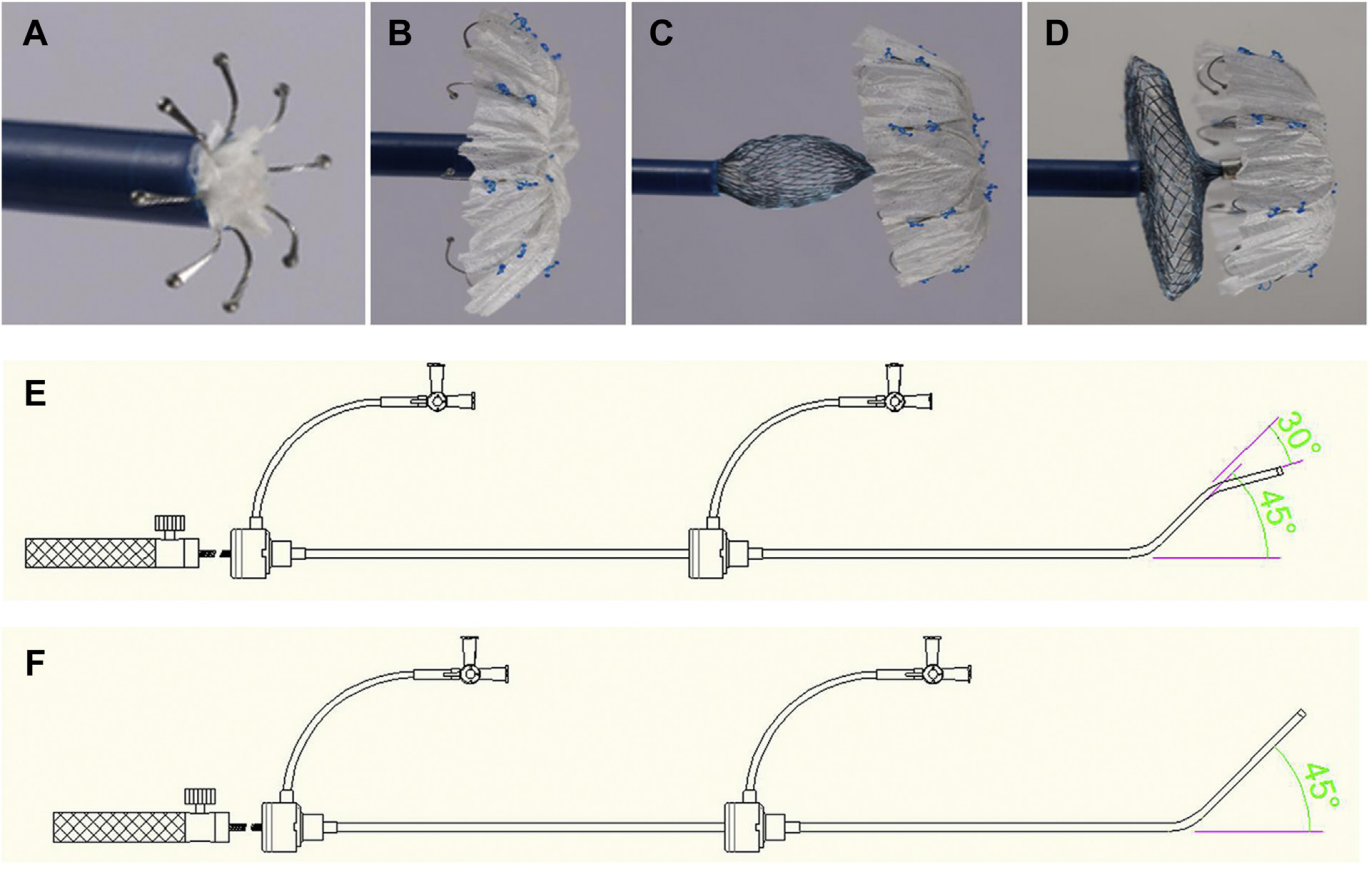
The Novel Disc-Like LAA Closure System **(A to D)** The implant in the process of being released. **(E)** The double-curve configuration delivery system. **(F)** The single-curve configuration delivery system. LAA = left atrial appendage.

The novel LAA closure system consists of 2 parts: the LAA closure implant and the delivery system. The implant is a self-expanding, nitinol-based device comprising a fixed umbrella and a blocking disc connected by a short central waist. The distal umbrella consists of 8 claws with single stable hooks and a polyethylene terephthalate membrane on the distal surface. The proximal disc is a 3-dimensional mesh structure with an isosceles design, which is sewn with polyethylene terephthalate fabric. The implants are divided into 2 types based on the difference between the diameter of the blocking disc and the fixed umbrella: the regular type (the disc is 4 mm larger than the umbrella) and the "small umbrella and large disc" type (the disc is 12 mm larger than the umbrella). The implant is available in 16 diameter sizes referring to the umbrella, ie, 15 to 33 mm. The delivery system comprises a double- or single-curve configuration delivery sheath (10-F in size), a loader, and a transmission cable (Figure 1).

**Figure 2:**
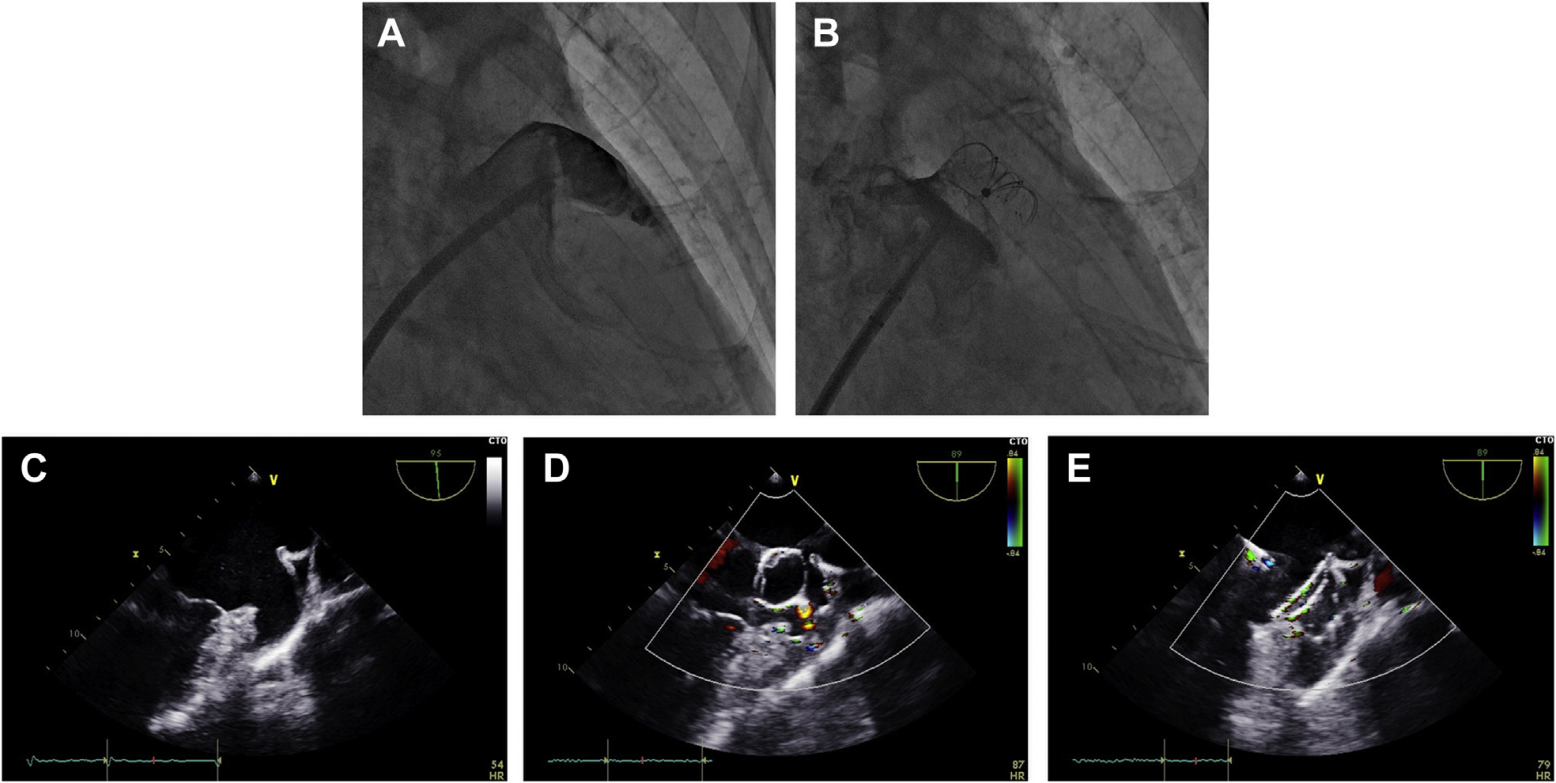
Well-Positioned Device in the LAA **(A)** LAA angiography before closure. **(B)** LAA angiography after closure. **(C)** Transesophageal echocardiography (TEE) examination before LAA closure. **(D)** A gentle tug test was performed to insure device stability. **(E)** TEE examination after LAA closure. Angiographic and TEE examinations suggested that the device was stable and completely occluded the LAA. Abbreviation as in Figure 1.

Under general anesthesia, implantation was performed via the femoral vein approach, guided by fluoroscopy, angiography, and continuous TEE. After transseptal puncture, intravenous heparin was given to obtain an activated clotting time >250 seconds. The diameters of the orifice and anchoring zone of the LAA were measured from LAA angiography in the right anterior oblique caudal projection. An upsizing of 4 to 8 mm above the largest orifice diameter obtained from intraprocedural imaging modalities was used. The distal umbrella was released into the LAA by stepwise pushing of the device out of the delivery sheath. Then, the sheath was withdrawn to expose the proximal disc so that it sealed the LAA ostium. Before device deployment, specific evaluation with angiography and TEE was performed following CODIS (Circumflex: The fixed umbrella is deployed behind the left circumflex coronary artery; Open: The fixed umbrella is fully opened; Disc: The blocking disc should be deformed; Insurance: A gentle tug test is performed to insure device stability; Sealing: The optimal sealing effect is achieved to reduce peridevice leakage [PDL]) criteria (Figure 2). If the position or stability is not ideal, the device would be completely retrieved and redeployed. PDL ≤3 mm was considered as immediate occlusion success.

### Follow-Up

All patients were prescribed anticoagulant agents or antiplatelet drugs after the LAA closure. Transthoracic echocardiography was performed before discharge to exclude the dislocation of device and acute device-related thrombus (DRT). Patients were followed up at 3, 6, and 12 months after LAA closure to evaluate outcomes. Successful occlusion is defined as TEE confirmation that there is no forward or reverse blood flow through the occluder, and the PDL does not exceed 3 mm, ie, grade 3 or above.^13^^,^^14^ TEE was scheduled at 3 months to evaluate device position, PDL, and DRT. Whether to perform TEE at 6 and 12 months was based on the 3-month follow-up result.

### Endpoint events

Serious adverse events (SAEs) that constituted the primary endpoint for safety included displacement or falling off of the occluder, cardiac tamponade requiring an intervention, other major bleeding requiring invasive treatment or blood transfusion, procedure-related stroke, and DRT. Primary efficacy endpoint events were composite endpoint events defined as hemorrhagic or ischemic stroke, systemic embolism, or cardiac or unexplained death. A single target value design was used to evaluate the noninferiority of the efficacy. Based on previous data with the same definition, the reported annual composite endpoint event rate after LAA closure in patients with NVAF was 3.0% to 8.3%.^9^^,^^10^^,^^15^ Clinical data from Chinese patients with AF showed that the incidence of stroke (not including systemic embolism and cardiac/unexplained death) was 6% to 13%.^16^ According to previous studies and clinical significance, the expected composite endpoint event rate (target value) was set at 3%, and the highest acceptable clinical annual incidence rate was no more than 8%. Secondary efficacy endpoint events included success rate of the operation, LAA closure effect, ischemic stroke, systemic embolism, and all-cause death.

### Statistical analysis

Continuous variables were presented as mean ± SD, and minimum and maximum values. Categorical variables were expressed as numbers (proportions). The procedure duration was compared using analysis of variance. The rate of SAEs between subgroups was compared using the chi square test, including single LAA closure, 1-stop LAA closure, and others. A value of *P* < 0.05 was considered statistically significant. The statistical criterion for success was the upper bound of a 2-sided 95% CI of <8% to ensure that the composite endpoint event rate was sufficiently low. The efficacy endpoints for the study were based on 1-sided tests. The Kaplan-Meier method was used for graphical assessment of time-related events. All statistical analyses were performed using SAS 9.4 (SAS Institute Inc).

## Results

### Baseline characteristics

**Figure 3:**
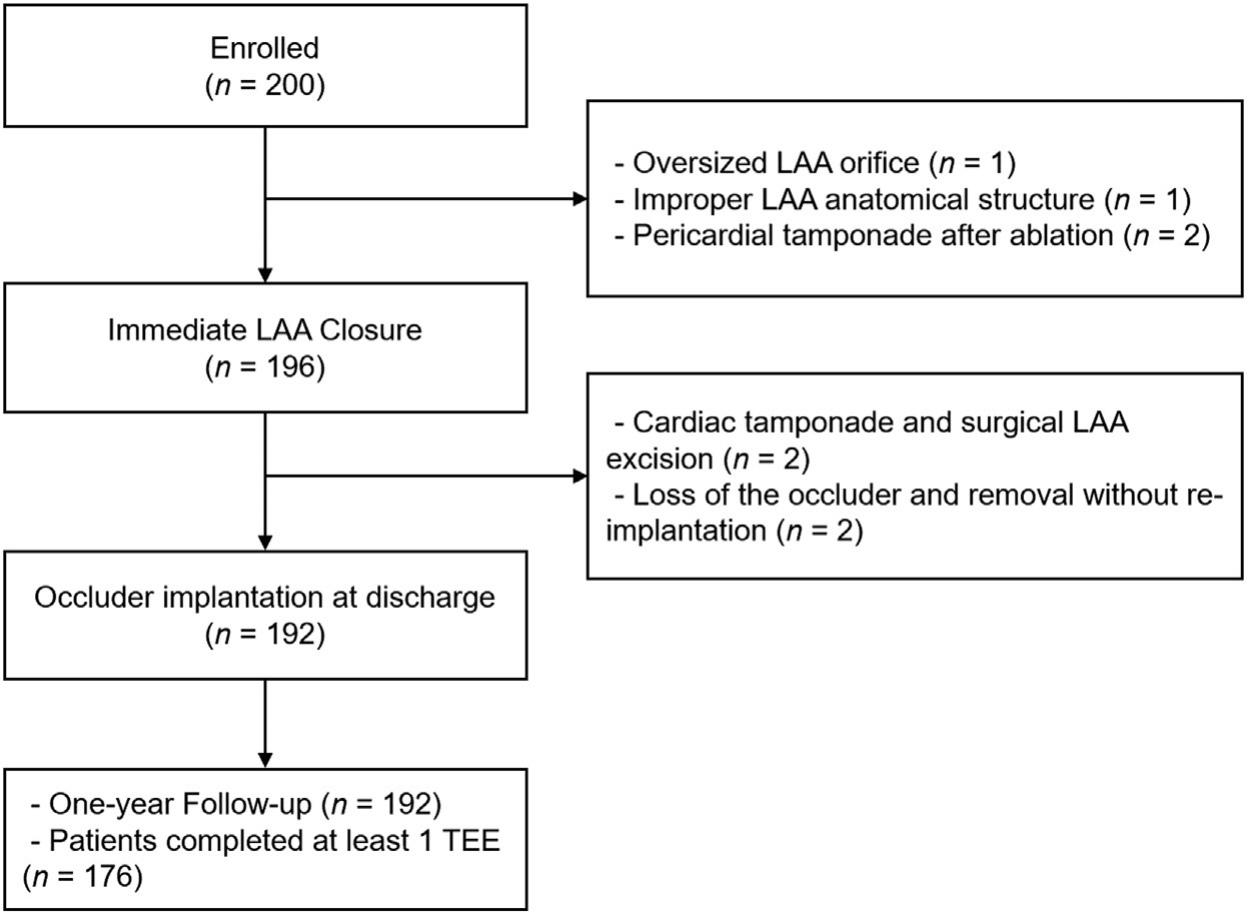
Study Flow Chart Between August 2018 and October 2019, a total of 200 patients with nonvalvular atrial fibrillation were enrolled in this study. The novel disc-like device was implanted in 196 patients. During the postoperative to discharge period, the occluder was removed from 4 patients caused by serious adverse events. The clinical follow-up was 100% complete, and the TEE follow-up was completed in 176 patients at 12 months. Abbreviations as in Figures 1 and 2.

There were 200 patients at 9 hospitals in China who met all inclusion and exclusion criteria and were enrolled in the study (Figure 3). The mean patient age was 68.3 years (range 38-89 years), and 56.5% were male. The most common risk factor for stroke was hypertension (63.5%), and 35% of patients previously had an ischemic stroke/transient ischemic attack. New York Heart Association functional classes I, II, and III accounted for 31.0%, 68.0%, and 1.0% of patients, respectively. The average CHA_2_DS_2_-VASc score was 3.5, and the average HAS-BLED score was 2.5. The baseline patient characteristics are shown in Table 1.

**Table 1:** Baseline Patient Characteristics (N = 200)

Clinical	
Age, y	68.3 ± 8.7
Men	113 (56.5)
Congestive heart failure	8 (4.0)
Hypertension	127 (63.5)
Age ≥65 y	133 (66.5)
Age ≥75 y	44 (22.0)
Diabetes mellitus	50 (25.0)
Previous history of TIA/stroke	70 (35.0)
Coronary artery disease	40 (20.0)
NYHA functional class	
I	62 (31.0)
II	136 (68.0)
III	2 (1.0)
CHA_2_DS_2_-VASc score	3.5 ± 1.4
2	56 (28.0)
3	58 (29.0)
4	42 (21.0)
5	21 (10.5)
6	18 (9.0)
7	3 (1.5)
8	2 (1.0)
HAS-BLED score	2.5 ± 1.2
0	11 (5.5)
1	37 (18.5)
2	55 (27.5)
3	71 (35.5)
4	18 (9.0)
5	6 (3.0)
6	2 (1.0)

Values are mean ± SD or n (%).

CHA_2_DS_2_-VASc = congestive heart failure, hypertension, age, diabetes, previous stroke/transient ischemic attack, vascular disease, female sex score; HAS-BLED = hypertension, abnormal renal/liver function, stroke, bleeding score; NYHA = New York Heart Association; TIA = transient ischemic attack.

### Implantation procedure

**Figure 4:**
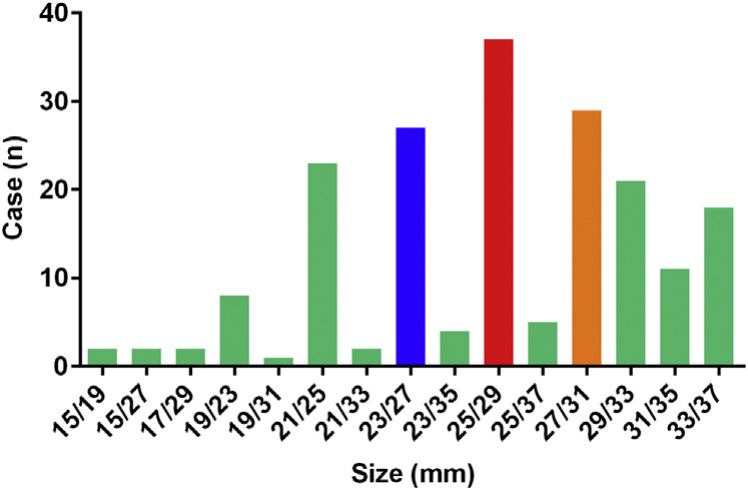
The Size Distribution of Successfully Implanted Occluders The size refers to the respective diameters of the umbrella and the disc. A total of 15 sizes were used, including 9 regular sizes (disc 4 mm larger) and 6 specific sizes (disc 12 mm larger).

**Figure 5:**
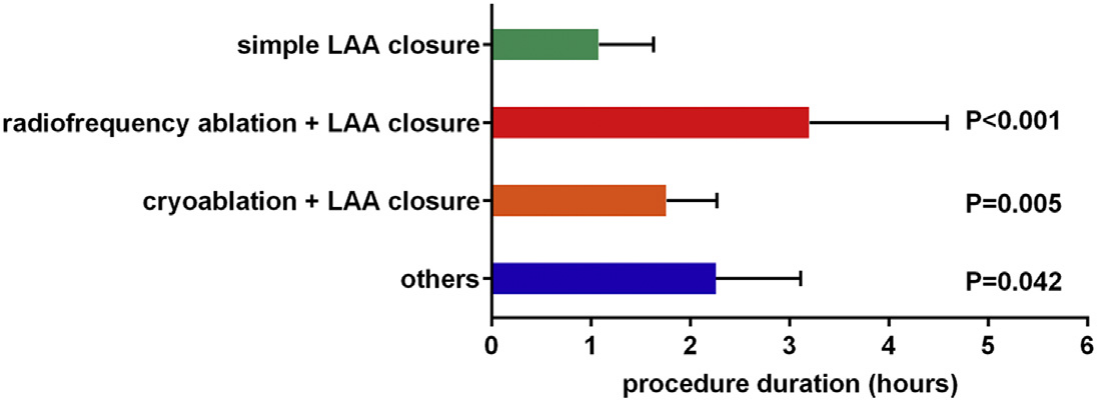
Duration of Different LAA Closure Procedures Others included 3 LAA closure + atrial septal defect closure, and 1 LAA closure + percutaneous coronary intervention. Abbreviation as in Figure 1.

The novel disc-like device was implanted in 196 patients (98.0%). The simple LAA closure, 1-stop operation of LAA closure combined with AF ablation, and others were performed in 63 (31.5%), 133 (66.5%), and 4 (2.0%) patients, respectively. All 196 patients were implanted with 1 occluder, and the remaining 4 patients did not receive implants. Among the patients who did not receive implants, 1 had an LAA orifice that was found to be larger than the upper limit (35 mm) of the protocol by angiography, 1 had an LAA structure that was not suitable for this device, and the other 2 patients had pericardial tamponade after AF ablation. The immediate success rate of occlusion was 100% (PDL ≤3 mm), of which 90.8% had no residual flow. Occlusion using the first device selected was successful in 148 patients. The 3 sizes with the highest proportion of implants were 25 mm/29 mm (19.3%), 27 mm/31 mm (15.1%), and 23 mm/27 mm (14.1%). The distribution of successfully deployed devices is shown in Figure 4. The mean procedure duration of simple LAA closure was 64.8 minutes, which was significantly less than that of the other groups (*P <* 0.05) (Figure 5). During the postoperative to discharge period, the occluder was removed from 4 patients caused by SAEs. At discharge, the final implantation success rate was 96.0%.

### Complications

There were SAEs in 9 patients (4.5%; 95% CI: 1.6%-7.4%) (Table 2). The procedure-related SAEs seemed to occur mainly in patients undergoing 1-stop LAA closure combined with AF ablation, although this difference did not reach statistical significance. Pericardial tamponade was clinically documented in 6 patients, and all occurred in 1-stop operations. Four of these patients were treated with pericardiocentesis, and 2 patients underwent surgical LAA excision. Because of loss or displacement of the occluder, it was removed in 3 patients: the first patient underwent occluder removal through the catheter without re-implantation; the second patient underwent occluder removal through the catheter with implantation of an alternative, long, plug-like device; and the third patient underwent surgery in parallel with LAA ligation. No patient experienced a procedure-related stroke or a major bleeding event. No acute DRT was observed before discharge. There were no procedure-related deaths.

**Table 2:** Procedure-Related Major Complications

	Simple LAA Closure (n = 63)	LAA Closure + AF Ablation (n = 133)	Total^a^ (N = 200)	*P* Value
Cardiac tamponade	0 (0.0)	6 (4.5)	6 (3.0)	0.205
Falling off of occluder	1 (1.6)	1 (0.8)	2 (1.0)	0.541
Displacement of occluder	1 (1.6)	0 (0.0)	1 (0.5)	0.321
Procedure-related stroke	0 (0.0)	0 (0.0)	0 (0.0)	—
Major bleeding	0 (0.0)	0 (0.0)	0 (0.0)	—
Acute DRT	0 (0.0)	0 (0.0)	0 (0.0)	—
Total	2 (3.2)	7 (5.3)	9 (4.5)	0.774

Values are n (%).

AF = atrial fibrillation; DRT = device-related embolization; LAA = left atrial appendage.

a There were 3 patients with LAA closure + atrial septal defect closure and 1 patient with LAA closure + percutaneous coronary intervention. None of the 4 patients had any complications.

### Follow-up results

During the 12-month follow-up period, the risk of primary composite endpoint events, including hemorrhagic or ischemic stroke, systemic embolism, and cardiogenic or unexplained death, was 1.6% (95% CI: 0.3%-4.5%). The upper bound of 4.5% was lower than the prespecified noninferiority margin of 8% that was predefined in the statistical analysis plan. Therefore, statistical noninferiority was achieved. One patient had an ischemic stroke, and 2 patients died suddenly from unknown causes. All-cause mortality was 2.6% (n = 5 of 192 patients). Three additional patients died of subdural hematoma with cerebral hernia caused by a tumble, pulmonary aspergillosis, and multiple organ failure. These deaths were deemed unrelated to the LAA closure device. After a 12-month follow-up period, 176 patients completed at least 1 TEE, and 173 patients (98.3%) had LAAs that were successfully occluded, with absence of flow or minimal flow around the device (PDL ≤3 mm). The 3 patients with residual flow >3 mm were the patients who underwent 1-stop LAA closure and ablation. No pulmonary venous obstruction or delayed DRT was found. The Central Illustration shows the clinical outcomes.

**Central Illustration:**
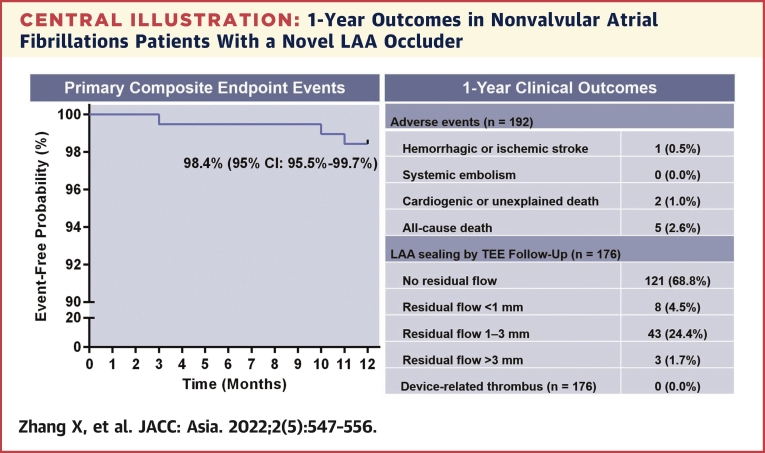
1-Year Outcomes in Nonvalvular Atrial Fibrillations Patients With a Novel LAA Occluder **(Left)** the Kaplan-Meier curve for primary composite endpoint events including hemorrhagic or ischemic stroke, systemic embolism, cardiogenic or unexplained death were shown. **(Right)** During 1-year of follow-up, the adverse events and the effect of left arterial appendage (LAA) sealing by transesophageal echocardiography (TEE) examination were shown. Values are n (%).

## Discussion

To our knowledge, this is the first study of 1-year follow-up results of patients with NVAF who underwent implantation of the novel disc-like LAA occluder. In this study, LAA closure with this device proved to be safe and effective and had a high immediate procedural success rate. Our follow-up results indicate that this device could be associated with encouraging clinical outcomes in preventing stroke.

The LAA anatomic shape restricts implantation of the LAA occluder to a certain extent. Therefore, different device forms are needed. The structure of the novel disc-like device is different from those of other devices. The WATCHMAN device is relatively long, mesh-like, and plug-shaped, so it is not suitable for patients with shallow or multilobed LAAs. In the PROTECT AF (Percutaneous Closure of the Left Atrial Appendage Versus Warfarin Therapy for Prevention of Stroke in Patients With Atrial Fibrillation) study, 32 patients (6.9%) did not receive LAA closure because the long, plug-shaped device did not match the shape of the LAA.^15^ For the same reason, 2 patients did not receive LAA closure in the PREVAIL (A Clinical Performance Evaluation of a New Medtronic Coronary Drug-coated Balloon Catheter for the Treatment of De novo Lesions, in-stent Restenosis and Small Vessel Disease in Coronary Arteries) study.^17^ With continued improvement in implantation techniques, the implantation success rate reached 98.5% in the EWOLUTION (Registry on WATCHMAN Outcomes in Real-life Utilization) study.^18^ Another LAA occluder, the Amplatzer Cardiac Plug device, comprises a distal cover and proximal disc connected by an articulated waist. It was reported that the procedural success rate with this device was 97.3%.^9^ However, neither of these devices are suitable for implantation if the LAA has an orifice >31 mm. Benefitting from its availability in various sizes, the LAmbre device, consisting of an umbrella and a cover connected by a short central waist, achieved 99.4% short-term procedural success.^10^ So et al^19^ retrospectively analyzed 964 cases of LAA closure. Among them, 27 patients (2.8%) with large LAAs were not suitable for receiving either the long, plug-shaped implant or the implant with the distal cover and proximal disc, and ultimately underwent implantation of the umbrella and cover device, with 24 cases (88.9%) successfully occluded (PDL ≤3 mm). Hence, the complementary advantages of different devices are particularly important in LAA closure. The novel disc-like device is somewhat similar to the umbrella and cover device, but its proximal component is a trapezoidal blocking disc, rather than a flat cover. The trapezoidal disc is partly inserted into the LAA, and can fit more LAA anatomic shapes and block the LAA more tightly without affecting the surrounding cardiac tissue. In addition, the anchors of the umbrella are inserted into the wall of the LAA and the U-shaped end is hooked into the pectinate muscles of the LAA, which are fixed to secure the device firmly. In this study, we achieved 98.0% immediate procedural success and 96.0% final implantation success. All patients receiving the occluder underwent successful short-term occlusion (PDL ≤3 mm). In contrast to the clinical trials described earlier, this study included a large number of patients (68.5%) who went on to undergo 1-stop LAA closure. Of these patients, 2 did not continue to LAA closure because of pericardial tamponade after AF ablation and not because of the device.

Cardiac tamponade is a serious complication of the LAA closure procedure. In the PROTECT AF trial, the operator's experience played an important role, reflected by the occurrence of this complication in 7.1% of the operator’s first 3 cases and in 4.4% of subsequent cases.^20^ The study of the implant with the distal cover and proximal disc reported cardiac tamponade in 3.6% of the patients.^21^ In our study, cardiac tamponade occurred in 3.0% of all the patients. Nevertheless, in our subgroup analysis, this complication did not occur in patients subject to single LAA closure, which occurred even less frequently than in the series with the umbrella and cover device (2.0%).^10^ During the procedures for implantation of either the long, plug-shaped device or the device with the distal cover and proximal disc, the delivery catheter often needs to be placed at the most distal end of the LAA, which could easily lead to LAA perforation. However, the deployment sequence of the new device is similar to that of the umbrella and cover device. LAA perforation is less likely to occur because the umbrella is released by gently pushing it forward to the landing zone. In this study, the cardiac tamponades all occurred during 1-stop LAA closure. This is because catheter ablation itself might cause cardiac tamponade. However, catheter ablation can lead to tissue edema, which makes the heart structure more susceptible to damage from LAA closure. In addition, after the recovery of sinus rhythm by catheter ablation, LAA contraction also increases the probability of operation injury. Moreover, the duration of 1-stop LAA closure is obviously longer than that of single LAA closure. Therefore, it is suggested that single LAA closure may be more tolerable.

The annual risk of primary composite endpoint events, including hemorrhagic or ischemic stroke, systemic embolism, and cardiogenic or unexplained death was 1.6% (95% CI: 0.3%-4.5%) in our study. It was reported that the composite endpoint rate in patients with NVAF after LAA closure was 3.0% to 8.3%.^9^^,^^10^^,^^15^ Although this result does not provide sufficient evidence to demonstrate the superiority of the novel system to other LAA closure systems, the results appear generally comparable to those reported for the long, plug-shaped device, the distal cover and proximal disc system, and the umbrella and cover system.^9^^,^^10^^,^^15^^,^^22^ In our 1-year follow-up, there was 1 patient (0.5%) with hemorrhagic or ischemic stroke and no patients with systemic embolism, representing significantly fewer events than expected according to the CHA_2_DS_2_-VASc scores.^11^ No DRT was found in this study during follow-up. In fact, DRT was most frequently detected within the first 3 months after LAA closure.^23^ However, Dukkipati et al^24^ reported that late DRT was detected in 27 of 1,504 patients (1.8%) at 12 months. Because the decision in this study on whether to perform subsequent TEE was based on the 3-month follow-up results, the incidence of late DRT may have been underestimated. Another possible reason that there was no DRT in this study is that the proportion of long-standing AF, an independent factor associated with DRT, was low after AF ablation.^23^ The immediate success rate of occlusion was 100% (PDL ≤3 mm), but there were 3 patients with 1-stop LAA closure and ablation who had residual flow >3 mm during follow-up. This may be caused by regression of tissue edema caused by ablation. The excellent efficacy of the novel system likely benefits from its unique structure: The distal umbrella pulls strongly on the proximal disc, designed as a wedge, combining the advantages of a plug and a cover and better fitting the LAA. This allows for a tighter seal of the LAA orifice and allows a larger contact area between the disc and the LAA orifice, preventing damage to the LAA endocardial surface. All of these advantages reduce residual flow, promote a faster healing response, and minimize DRT effectively. Actually, there are many other factors that affect the efficacy of LAA closure, including antithrombotic therapy.^25^^,^^26^ As there have been no randomized controlled trials of antithrombotic therapy in LAA closure, this must be further investigated.

### Study limitations

This is a first-in-human study for the novel disc-like occluder. Because most operators are not familiar with this novel device, it could increase the difficulty of implantation and the risk of adverse events. In addition, there is no control group included in this study. The late DRT may be underestimated because of insufficient TEE follow-up at 12 months. This study also had a small sample size with a medium-term follow-up. Therefore, it is not sufficient to allow conclusions to be drawn about the long-term efficacy and safety of this device in preventing stroke in patients with NVAF. Thus, prospective, multicenter, randomized, controlled (novel disc-like occluder vs oral anticoagulant agents) clinical trials are needed to further confirm the long-term safety and efficacy of this novel device.

## Conclusions

LAA closure with the novel disc-like device shows high procedural success, satisfactory safety, and encouraging efficacy for stroke prevention in patients with NVAF. Compared with 1-stop operation of LAA closure combined with AF ablation, single LAA closure may be more tolerable.

Perspectives **COMPETENCY IN MEDICAL KNOWLEDGE:** In patients with NVAF, most thrombosis originates from the LAA. The preliminary study suggested that percutaneous LAA closure with the novel disc-like occluder device is feasible. This prospective, multicenter, registry-based clinical study with 1-year follow-up showed a low ischemic stroke rate with a small number of complications and that 1-stop operation of LAA closure combined with AF ablation may be relatively poorly tolerated.

**TRANSLATIONAL OUTLOOK:** This was a single-arm study and reported the midterm clinical outcomes for LAA closure with a novel disc-like occluder. Further prospective, multicenter, randomized, controlled clinical trials are needed to confirm the long-term efficacy and safety of the novel device.

## Funding Support and Author Disclosures

This work was supported by grants from the National Key R& D Program of China (No. 2020YFC1107800 to Dr Zhou), and the Shanghai Clinical Research Center for Interventional Medicine (No. 19MC1910300 to Dr Ge). The authors have reported that they have no relationships relevant to the contents of this paper to disclose.
